# Effect of Cold Storage on the Quality of *Psyttalia incisi* (Hymenoptera: Braconidae), a Larval Parasitoid of *Bactrocera dorsalis* (Diptera: Tephritidae)

**DOI:** 10.3390/insects12060558

**Published:** 2021-06-16

**Authors:** Jia Lin, Deqing Yang, Xuxing Hao, Pumo Cai, Yaqing Guo, Shuang Shi, Changming Liu, Qinge Ji

**Affiliations:** 1 Institute of Beneficial Insects, Plant Protection College, Fujian Agriculture and Forestry University, Fuzhou 350002, China; Linjia@fafu.edu.cn (J.L.); 3200231054@fafu.edu.cn (D.Y.); 1200203004@fafu.edu.cn (X.H.); caipumo@fafu.edu.cn (P.C.); 3190231007@fafu.edu.cn (Y.G.); 1190203017@fafu.edu.cn (S.S.); cmliu@fjau.edu.cn (C.L.); 2 Key Laboratory of Biopesticide and Chemical Biology, Ministry of Education, Fuzhou 350002, China; 3 State Key Laboratory of Ecological Pest Control for Fujian and Taiwan Crops, Fuzhou 350002, China; 4 Department of Horticulture, College of Tea and Food Science, Wuyi University, Wuyishan 354300, China

**Keywords:** *Psyttalia incisi*, oriental fruit fly, cold storage, emergence rate, quality, reproduction

## Abstract

**Simple Summary:**

Biological control programs primarily rely on the mass-release of high-quality bioagents in order to successfully suppress pests. However, producing such bioagents on a large scale and within a short timeframe or in a single step is extremely difficult. Therefore, it is important to consider methods that could increase the shelf life and help to synchronize the release schedule of bioagents reared in different batches. In the present study, we determined the effects of various cold storage protocols on the emergence and quality of *Psyttalia incisi*, a larval parasitoid of *Bactrocera dorsalis*. Our results indicated that there were no negative impacts on the emergence parameters and adult quality when late-age *P. incisi* pupae were stored at 13 °C for 10 or 15 d. This information is valuable in facilitating the mass-rearing of *P. incisi* and helping to improve the efficiency of biological control programs using *P. incisi* against *B. dorsalis.*

**Abstract:**

*Psyttalia incisi* (Silvestri) is the dominant parasitoid against *Bactrocera dorsalis* (Hendel) in fruit-producing regions of southern China. Prior to a large-scale release, it is important to generate a sufficient stockpile of *P. incisi* whilst considering how best to maintain their quality and performance; cold storage is an ideal method to achieve these aims. In this study, the impacts of temperature and storage duration on the developmental parameters of *P. incisi* pupae at different age intervals were assessed. Then, four of the cold storage protocols were chosen for further evaluating their impacts on the quality parameters of post-storage adults. Results showed that the emergence rate of *P. incisi* was significantly affected by storage temperature, storage duration, and pupal age interval and their interactions. However, when late-age *P. incisi* pupae developed at a temperature of 13 °C for 10 or 15 d, no undesirable impacts on dry weight, flight ability, longevity, reproduction parameters of post-storage adults, emergence rate, or the female proportion of progeny were recorded. Our findings demonstrate that cold storage has the potential for enhancing the flexibility and effectiveness of the large-scale production and application of *P. incisi.*

## 1. Introduction

*Batrocera dorsalis* (Hendel) (Diptera: Tephritidae) is a notorious pest of economic importance, largely due to its traits of polyphagia, superior dispersal ability, outstanding climate adaptability, and high fecundity [1]. Over the last two decades, this fly has spread into many tropical and subtropical regions due to both human transportation and adult fly migration, causing considerable damage to commercial fruits and horticultural products as well as the associated import and export trade [2,3]. At present, the primary strategy for suppressing this pest involves spraying chemical insecticides, either alone or in combination with food-based lures [4]. However, there are numerous problems associated with this strategy, including insecticide resistance, environmental depredation, and effects on food safety, which has led to pressure for an alternative strategy to be developed [5,6]. Biological control, which has the advantages of being long-lasting and environmentally friendly, has recently gained much attention and is regarded as the prime alternative tactic against *B. dorsalis* populations [7].

*Psyttalis incisi* (Silvestri) (Hymenoptera: Braconidae: Opiinae) is a solitary opiine endoparasitoid, whose preferential host is the early larval instar of *B. dorsalis* [8]. It has been reported that *P. incisi* exist naturally and occupy a dominant proportion (77.6%) of the parasitic wasp population against *B. dorsalis* in fields of Zhangzhou City, Fujian Province, China, and contributed a limited reduction in *B. dorsalis* populations [9]. Hence, *P. incisi* is a highly suitable bioagent for biological control programs against *B. dorsalis* in that region of China. However, in order for such programs to be effective, millions of parasitoids need to be first produced and then transported into the affected areas. In order to improve the ease at which this can be achieved, it is important to consider methods by which the shelf life of *P. incisi* can be increased. This will help to ensure a sufficient stockpile of parasitoids and allow releases to be appropriately timed given their physiological characteristics.

Exposing bioagents to low temperatures can extend their developmental duration and is particularly valuable for the inundative biological control program which entailed a large number of biocontrol agents [10]. However, keeping parasitoids at a sub-ambient temperature can result in cold injuries and excessive consumption of energy reserves, resulting in undesirable effects on the quality and quantity of post-storage parasitoids [11]. Hence, to minimize losses in the performance of parasitoids after undergoing storage, packaging, and shipment, recent research has focused on optimizing the tradeoffs between quality and the cold storage protocol [12,13,14]. For braconid parasitoids against tephritid pests, the impacts of cold storage have been evaluated in several species, such as *Psyttalia humilis* (Silvestri), *Psyttalia ponerophaga* (Silvestri), *Fopius arisanus* (Sonan), and *Diachasmimorpha longicaudata* (Ashmead) (Hymenoptera: Braconidae); these works have provided a lot of valuable information for facilitating the practical application of biological control programs [15,16,17]. However, to date, cold storage has never been investigated as an auxiliary approach for the large-scale rearing and release of *P. incisi*. Owing to the vital role of *P. incisi* in the management of *B. doralis*, it is crucial to optimize the flexibility and effectiveness of large-scale production and mass-release programs of *P. incisi* through cold storage techniques.

In the present study, we first determined the effects of various storage temperatures (4, 7, 10, and 13 °C) and exposure durations (10, 15, 20, and 25 days) on the emergence rate of *P. incisi* at different pupal age intervals within parasitized *B. dorsalis* puparia. Further experiments were then carried out to examine the dry weight, flight ability, longevity, and reproduction parameters of parental *P. incisi* (G1) along with emergence rates, and the female proportion of progeny (G2).

## 2. Materials and Methods

### 2.1. Insect Colonies 

*Bactrocera dorsalis* and *P. incisi* were initially collected from orchards in and around Fujian institutes of Tropical Crops (117°30′58.95″ E, 24°37′31.37″ N, and 26 m altitude), in Zhangzhou City, Fujian Province, China in 2004. These orchards are composed of ‘Pearl’ guava (*Psidium guajava* L.), carambola (*Averrhoa carambola* L.), and wax apple (*Syzygium samarangense*), the annual average temperature of this area was 21 °C and annual average rainfall was 1603 mm [9]. *Bactrocera dorsalis* and *P. incisi* were then identified in the laboratory [18], and these vouchers were deposited in the UN (China) Center for Fruit Fly Prevention and Treatment, Fujian Agriculture and Forestry University. *Bactrocera dorsalis* were permitted to oviposit in a plastic bottle that was neatly pierced with holes; eggs were collected and transferred to a tray containing a mill feed diet for larval development, prepared in accordance with Chang et al. [19]. Puparia were collected from the bottom of the tray and transferred into a gauze cage (30 × 30 × 30 cm^3^) until emergence. Fly adults were provided with a diet of yeast extract and sugar (1:3, wt:wt) and water. For the rearing of *P. incisi*, excessive numbers of second-instar *B. dorsalis* larvae were transferred to an oviposition plate (diameter: 9 cm, high: 0.5 cm) which was covered with 80 mesh net to prevent larvae from escaping. Then, two oviposition plates were provided to 500 pairs of *P. incisi* adults within a cage for 24 h to avoid super-parasitism. Honey and water were provided for *P. incisi* adults. *Bactrocera dorsalis* and *P. incisi* used in the experiments were reared under the controlled conditions of 25 ± 1 °C, 65 ± 5% relative humidity (RH), and a 12:12 h (L:D) photoperiod.

### 2.2. Effects of Storage Temperature, Storage Duration and Pupal Age Interval on the Emergence Parameters of P. incisi

Newly-formed *B. dorsalis* puparia were collected daily, with parasitized and unparasitized puparia distinguished by several characteristics as described in Wang and Messing [20] and Danne et al. [15]. Briefly, for parasitized puparia, the major characteristics included oviposition scars on the cuticle, a gap inside the fly pupa caused by consumption of the fly body by a parasitoid larva, and being relatively smaller and browner. For unparasitized puparia, the appendages of the fly could be clearly observed through the cuticle under a microscope.

Based on a preliminary experiment that identified the immature stage of *P. incisi* (through dissecting puparia), parasitized *B. dorsalis* puparia were incubated at 25 °C for 3, 6, and 9 days for *P. incisi* to develop into prepupae, middle-age pupae (some appendages, the shape of abdomen and thorax are observable but without any color; eye and ocelli are rufous), and late-age pupae (body is tawny, eye and ocelli are dark, mouth is dark brown), respectively. The three developmental stages were subsequently stored in four incubators (PRX-25013, Safu, Ningbo, China) set at constant low temperatures (4, 7, 10, and 13 °C, respectively) for four durations (10, 15, 20, and 25 d), all at 75% RH. A control group comprised parasitized puparia that developed in an incubator set at 25 °C and 75% RH until emergence. For each treatment and control, Petri dishes contained 30 parasitized puparia each were prepared. To ensure a ventilated condition to avoid the outbreak of pathogens, gauze was used to cover the dishes. Dishes were randomly assigned to the various treatment groups and inspections of each incubator were performed daily (for 30 s) to observe whether *P. incisi* adults had emerged during the cold exposure period. After cold exposure, treatments were held under control conditions and the number and sex of emerged *P. incisi* adults were recorded daily. All unemerged puparia of each treatment were dissected 5 days after the last *P. incisi* emerged or 15 days after cold treatment protocol (for those treatments without *P. incisi* emerged). A total of nine replicates were performed for this experiment.

### 2.3. Effects of Pupal Cold Storage on Quality of P. incisi Adults

To determine the effects of cold exposure on the G1 quality and G2 emergence parameters, four pupal cold storage protocols that did not have significant negative impacts on the emergence rate of *P. incisi* were selected for further experiments based on the results of Experiment 2.2. After the cold storage protocol, this series of bioassays were conducted under controlled conditions of 25 ± 1 °C and 65 ± 5% RH.

The four cold storage protocols were as follows:

Cold storage 1 (CS1): late-age *P. incisi* pupa (9-day-old parasitized *B. dorsalis* puparia) stored at 13 °C for 10 days;

Cold storage 2 (CS2): middle-age *P. incisi* pupa (6-day-old parasitized *B. dorsalis* puparia) stored at 13 °C for 10 days;

Cold storage 3 (CS3): late-age *P. incisi* pupa (9-day-old parasitized *B. dorsalis* puparia) stored at 13 °C for 15 days;

Cold storage 4 (CS4): middle-age *P. incisi* pupa (6-day-old parasitized *B. dorsalis* puparia) stored at 13 °C for 15 days.

#### 2.3.1. Dry Weight

Parasitized puparia were transferred to a plastic bowl within a gauze cage. Parasitoids that had emerged within 24 h without foraging any food were used in this bioassay. For each treatment, 1 group of 50 females and 1 group of 50 males were respectively placed inside a total of 6 centrifuge tubes. All adults were kept at –20 °C for 20 min and were then dried in an oven at 60 °C for 48 h. Subsequently, the gross dry weight of a cohort of 50 females or males from each group was measured using a semimicro balance (CP225D, accuracy of 0.01 mg, Sartorius, Göttingen, Germany). Nine replicates were conducted in this experiment.

#### 2.3.2. Flight Capacity

Fifty newly-emerged (<24 h) females and 50 newly-emerged males were caught using 5 mL centrifuge tubes. These centrifuge tubes were held at 4 °C for 10 s to immobilize the adult wasps. Subsequently, two tubes each of females and males were placed into a hollow black cylinder that had been placed in the center of a cage and the lids of these tubes were opened and no longer closed to allow them to escape. Talc powder was uniformly daubed around the interior of the black cylinder to ensure that adults could not climb out. To provide illumination, a 30 W fluorescent light was directed at the top of the cage from a distance of 20 cm. The collection of fliers was performed every 12 h. The experiment was performed until all parasitoid wasps were dead. The flight capacity was calculated as (the total number of *P. incisi* flew out the hollow black cylinder / the total number of *P. incisi* used) × 100%. For each treatment, nine replicates of this experiment were conducted.

#### 2.3.3. Longevity and Reproductive Parameters

Pairs of *P. incisi* adults that had emerged within 24 h were confined in individual centrifuge tubes to observe mating behavior; this ensured that all adults used in this experiment were mated. Subsequently, each pair of *P. incisi* adults were transferred into a plastic jar (diameter: 15 cm, high: 10 cm) and the top was wrapped with gauze for ventilation. Honey and water were provided: honey was daubed on the gauze and water was absorbed in a sponge inside the plastic jar. Excessive numbers of second-instar *B. dorsalis* larvae were transferred to a small oviposition plate (diameter: 3.5 cm, high: 0.5 cm) that was covered with 80 mesh net, and then a small oviposition plate was provided for *P. incisi* adults to parasitize for 24 h. The larvae were refreshed daily until the female had died. After parasitism, larvae were reared on artificial diets until pupation. Larvae and pupae that died during development were removed for further observation and dissection under a microscope to determine whether they had been parasitized. The number of both sexes of the progeny of each treatment and control were documented daily. The pre-oviposition period, oviposition period, post-oviposition period, longevity of G1, and emergence rate as well as the female proportion of G2 were recorded for each treatment and control. A total of 15 replicates were performed for each treatment.

### 2.4. Statistical Analysis

All data were analyzed after checking that the data were normally distributed and there was homogeneity of variances (SPSS Inc., Chicago, IL, USA). Percentage data were arcsine square-root-transformed for further statistical analysis; however, untransformed data are presented in tables. The effects of storage temperature, storage duration, pupal age interval, and their interactions on emergence and proportion of female G1 were analyzed by univariate three-way ANOVA (generalized linear model, GLM). One-way ANOVA was conducted to analyze the effects of the pupal cold storage protocol on dry weight, flight ability, longevity, and reproduction parameters of G1 *P. incisi* adults and the emergence rate and female proportion of G2. Differences between treatments and control were assessed using a one-way ANOVA with Tukey’s honestly significant difference (HSD) test (*p* < 0.05) for multiple mean comparisons.

## 3. Results

### 3.1. Effects of PupalCold Storage on the Emergence Parameters of P. incisi

#### 3.1.1. Emergence Rate

A significant difference was observed in the emergence rate of *P. incisi* among storage temperature (*F*_3, 432_ = 1286.705, *p* < 0.001), storage duration (*F*_3, 432_ = 426.532, *p* < 0.001), pupal age interval (*F*_2, 432_ = 196.2910, *p* < 0.001), storage temperature × storage duration (*F*_9, 432_ = 12.342, *p* < 0.001), storage temperature ×pupal age interval (*F*_6, 432_ = 68.522, *p* < 0.001), storage duration × pupal age interval (*F*_6, 432_ = 6.573, *p* < 0.001), and their interactions (*F*_18, 432_ = 3.881, *p* < 0.001).

Overall, the emergence rate of *P. incisi* pupae of the same age decreased as temperature decreased and storage duration increased. Regardless of pupal age interval and storage duration, *P. incisi* pupae stored at 4, 7, and 10 °C exhibited significantly lower emergence rates compared to the control group. However, no significant differences were observed for middle-age *P. incisi* pupae stored at 13 °C for 10 days (*p* = 1.000) and 15 days (*p* = 0.343) as well as late-age *P. incisi* pupae stored at 13 °C for 10 days (*p* = 1.000) and 15 days (*p* = 0.779) in comparison with the control group. Surprisingly, after being subjected to the same cold storage protocol of 4, 7, and 10 °C, middle-age *P. incisi* pupae presented the highest emergence rate compared to the prepupae and late-age pupae (Table 1).

#### 3.1.2. Female Proportion

The proportion of females was not affected by storage temperature (*F*_3, 253_ = 0.469, *p* = 0.704), storage duration (*F*_3, 253_ = 0.698, *p* = 0.554), pupal age interval (*F*_2, 253_ = 0.027, *p* = 0.973), storage temperature × storage duration (*F*_6, 253_ = 0.490, *p* = 0.816), storage temperature × pupal age interval (*F*_5, 253_ = 0.093, *p* = 0.993), storage duration × pupal age interval (*F*_6, 253_ = 0.087, *p* = 0.998), or their interactions (*F*_3, 253_ = 0.069, *p* = 0.976). There was no significant difference between the control group and all treatments (Table 2).

### 3.2. Effects of Pupal Cold Storage on the Quality of P. incisi Adults

#### 3.2.1. Dry Weight

Pupal cold storage lead to significant impacts on the dry weight of both sexes of post-storage *P. incisi* adults according to one-way ANOVA (female: *F*_4, 40_ = 36.795, *p* < 0.001; male: *F*_4, 40_ = 22.323, *p* < 0.001). Furthermore, based on the result of Tukey’s HSD test, except for the cold storage 4 (CS4) protocol (female: *p* < 0.05; male: *p* < 0.05), all other pupal cold storage treatments did not differ from the control for the dry weight of both sex adults (Table 3).

#### 3.2.2. Flight Capacity

The flight capacity of post-storage *P. incisi* adults was significantly affected by the cold storage protocol (female: *F*_4, 40_ = 3.520, *p* < 0.05; male: *F*_4, 40_ = 2.926, *p* < 0.05). CS4 lead to the lowest flight capacity of both sex adults and was significantly inferior to the control (female: *p* < 0.05; male: *p* < 0.05) (Table 3).

#### 3.2.3. Longevity

**Figure 1:**
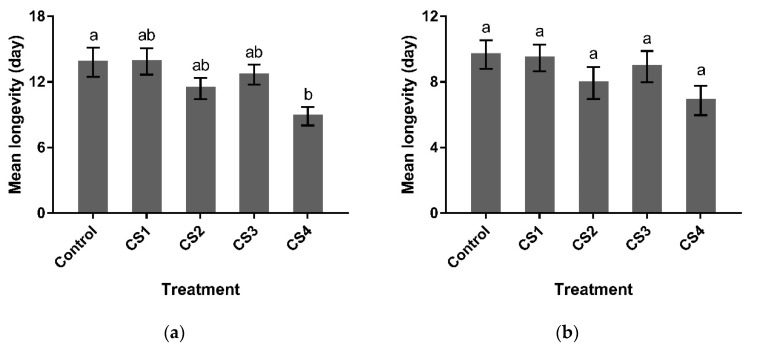
Longevity of G1 *P. incisi* post-storage females (**a**) and males (**b**) that had been subjected to different pupal cold storage treatments. CS1: cold storage 1, late-age pupae stored at 13 °C for 10 d; CS2: cold storage 2, middle-age pupae stored at 13 °C for 10 d; CS3: cold storage 3, late-age late pupae stored at 13 °C for 15 d; CS4: cold storage 4, middle-age pupae stored at 13 °C for 15 d. Control means *P. incisi* pupae developed at 25 °C. Bars topped with the same letter do not differ significantly (*p* > 0.05) according to Tukey’s HSD test (one-way ANOVA) (*n* = 15).

**Figure 2:**
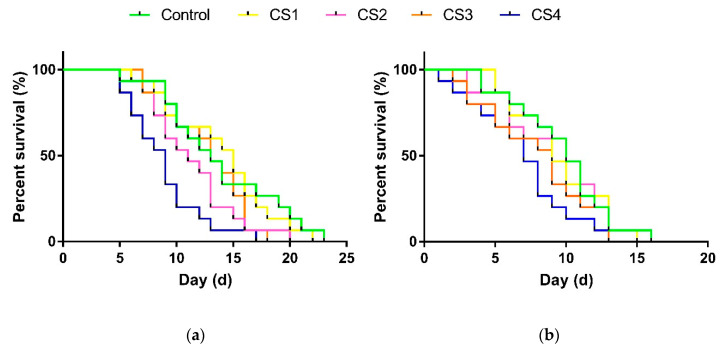
Survival curves of *P. incisi* post-storage female (**a**) and male (**b**) that had been subjected to different pupal cold storage treatments. CS1: cold storage 1, late-age pupae stored at 13 °C for 10 d; CS2: cold storage 2, middle-age pupae stored at 13 °C for 10 d; CS3: cold storage 3, late-age late pupae stored at 13 °C for 15 d; CS4: cold storage 4, middle-age pupae stored at 13 °C for 15 d. Control means *P. incisi* pupae developed at 25 °C (*n* = 15).

For female parasitoids, pupal cold storage had significant effects on longevity (female: *F*_4, 70_ = 3.795, *p* < 0.01), and a remarkable reduction in female longevity was observed for CS4 (*p* < 0.05). However, the longevity of males was not significantly influenced by different treatments (*F*_4, 70_ = 1.669, *p* = 0.167) (Figure 1 and Figure 2).

#### 3.2.4. G1 Reproductive Performance and G2 Emergence Parameters

Pre-oviposition period (*F*_4, 70_ = 0.353, *p* = 0.841), post-oviposition period (*F*_4, 70_ = 2.128, *p* = 0.086), G2 emergence rate (*F*_4, 70_ = 0.412, *p* = 0.799), and G2 female proportion (*F*_4, 70_ = 1.864, *p* = 0.127) were not significantly affected by pupal cold storage. However, significant effects were observed for total offspring per female (*F*_4, 70_ = 15.245, *p* < 0.001), daily offspring (*F*_4, 70_ = 3.625, *p* < 0.05), and oviposition period (*F*_4, 70_ = 3.893, *p* < 0.001). The highest total offspring produced by a female was in the control group, and there was a significant difference between the control in comparison to females subjected to CS2 (*p* < 0.05) and CS4 (*p* < 0.01). Furthermore, CS4 resulted in significantly lower daily offspring (*p* < 0.05) and a shorter oviposition period (*p* < 0.05) than the control (Table 4 and Table 5).

## 4. Discussion

The utilization of opiine parasitoids, especially *F. arisanu*, has successfully alleviated the serious damage of *B. dorsalis* to fruit products in Hawaii, which inspired other regions to adopt a biological control program for managing this pest [21]. However, today, *F. arisanu* has only been recorded in Zhanjiang City, Guangdong Province in China [22], which means that *F. arisanu* may not be able to be the dominant bioagent against *B. dorsalis* in some regions of China. Furthermore, considering the various ecological conditions in different regions of mainland China, it is indispensable to dig local parasitoid resources to develop the most suitable local biological control programs against *B. dorsalis*. Previous research in Zhangzhou City, Fujian Province, China, indicated that there were four parasitic wasp species against *B. dorsalis*, including *P. incisi, Pachycrepoideus vindemmiae* (Rondani), *Pachycrepoideus vindemmiae* (Rondani), and *Spalangia endius* (Walker) (Hymenoptera: Pteromalidae), of which P. *incisi* occupied a dominant proportion (77.6%) of this parasitic wasp population [9]. Therefore, P. *incisi* is a highly suitable bioagent for biological control programs against *B. dorsalis* in that region of China.

The present study is the first, to our knowledge, that has aimed to optimize the cold storage protocol of *P. incisi* to improve the efficiency and flexibility of biological control programs against *B. dorsalis*. Our results demonstrate that storage temperature, storage duration, pupal age interval, storage temperature × storage duration, storage temperature ×pupal age interval, and storage duration × pupal age interval and their interactions have significant impacts on the viability of immature *P. incisi*. However, when late-age *P. incisi* pupae were subjected to CS1 and CS3, the emergence parameters of both G1 and G2 progeny, and G1 quality parameters (including flight ability, dry weight, longevity, and reproduction parameters) did not differ significantly from the control treatment.

The essence of cold storage is to utilize a sub-optimum temperature to prolong the developmental time of a bioagent whilst maintaining its quality and effectiveness against a pest [23]. Cold storage protocols are extremely valuable for inundative biological control, given that they require large-scale production and the release of huge numbers of bioagents. However, modulating the development of bioagents via cold storage can pose lethal and sub-lethal effects to their survivorship [24,25]. In the present study, a reduction in the storage temperature or an extension of the storage period resulted in a decrease in the emergence rate of *P. incisi*. This is consistent with previous research on the cold storage of two other braconid parasitoids that are used against *B. dorsalis*, namely *F. arisanus* and *D. longicaudata*; these studies found that the viability of parasitized pupae was gradually reduced as the temperature decreased or the storage period was extended [16,17]. In addition, we interestingly found that middle-age *P. incisi* pupae exhibited superior performance in the emergence rate in comparison to prepupae and late-age pupae subjected to the same cold storage protocols of 4, 7, or 10 °C. Similarly, a study on *Encarsia formosa* (Gahan) (Hymenoptera: Aphelinidae) indicated that both early- and late-stage pupae exhibited inferior tolerance to low temperatures in comparison with mid-stage pupae, with the mid-stage pupae exhibiting higher survival rates [26].

Although cold storage clearly has advantages in enhancing the efficiency and flexibility of mass-rearing and release programs, the undesirable effects on the quality of post-storage insects are of concern. A significant amount of research has emphasized that subjecting bioagents to cold conditions can result in cold stress and excessive consumption of energy reserves, thereby influencing their quality parameters and their successful application under field conditions [11,27]. Our results demonstrated that the cold storage of *P. incisi* pupae was deleterious to the dry weight of post-storage adults. Lins et al. reported that the body mass loss was directly proportional to the extension in the storage period when pre-pupae of *Praon volucre* (Haliday) (Hymenoptera: Braconidae) were stored at 5 °C [28]. These weight losses during cold storage may chiefly result from the immature parasitoid wasps consuming a body of energy and lipid reserves to ensure survival during the period of low temperature. Excessive lipid loss is fatal to parasitoid adults as they cannot synthesize lipids by themselves, and thereby perform a trade-off between survivorship and fecundity, in turn affecting their lifespan [29,30,31]. In our study, the longevity of female *P. incisi* that emerged from middle-age pupae stored at 13 °C for 15 days was significantly shorter. This is in accordance with results found in *D. longicaudata* [17], which revealed that females that emerged from parasitized *B. doralis* pupae stored below 8 °C had a shorter lifespan than the control, regardless of the storage period. Likewise, the cold storage of other braconid wasps, such as *Aphidius ervi* (Haliday), *Aphidius* *picipes* (Nees), and *Bracon hebetor* (Say) (Hymenoptera: Braconidae), during the pupal stage, indicated a similar tendency in the longevity of post-storage adults [32,33,34].

Dispersibility, one of the most important quality parameters for parasitoids when considering the various, complex, and harsh field conditions they need to withstand whilst host-seeking, hiding from predators, and searching for resting places, is particularly vulnerable to the effects of low-temperature storage. In fact, the neuro-muscular dysfunction that can be induced by cold storage directly affects the bioagent’s ability to disperse, and is a major obstacle to the practical application of post-storage insects in the field [10,35]. In concordance with this, our results demonstrated that subjecting middle-age *P. incisi* pupae to 15 days of cold storage at 13 °C resulted in the emerged adults displaying inferior flight ability. A study on *E. formosa* and *Encarsia eremicus* (Rose) (Hymenoptera: Aphelinidae) indicated that increasing the cold exposure period of pupae resulted in a reduction in the flight capacity of the emerged adults [26]. Similarly, *P. volucre* pre-pupae developed at a sub-ambient temperature of 5 °C exhibited lower flight capacity than the control group [28].

In addition to dispersibility, the reproductive system of bioagents is extremely susceptible to sub-optimum conditions. In our study, for middle-age *P. insici* pupae subjected to cold storage at 13 °C for 10 or 15 days, the number of progeny produced by post-storage adults was remarkably decreased. This is consistent with previous research on braconid parasitoids, whereby a reduction in fecundity was strongly associated with low temperature and storage period [28,33,36]. Furthermore, the reproductive parameters of *P. incisi* that originated from the Zhangzhou region showed a significant difference to the Hawaii *P. incisi* strain [37]. Liang et al. [38] indicated that there was a certain difference between two geographic *P. incisi* populations by using random amplified polymorphic DNA analysis, this may account for the remarkable difference in reproductive parameters between these two strains.

The viability of mass-release biocontrol programs is largely determined by the ability to generate sufficient reserves of the bioagent in a relatively short timeframe. Furthermore, flexibility is also required in both the rearing and release schedules to deal with unforeseen factors such as adverse weather and transportation delays. As such, the cold storage technique can help mitigate these problems [39] and is increasingly undertaken as a support approach in classical biological control programs [11]. Nonetheless, the adverse effects induced by sub-ambient temperature remarkably reduce the performance of post-storage bioagents. This consequently leads to challenges in balancing the logistics of the release program with field performance. In order to minimize the losses in quantity and fitness, recent studies have primarily concentrated on optimizing the cold storage protocol of the bioagent. As such, our present study aims to provide a foundation for optimizing the cold storage technique of *P. incisi* and our results demonstrate that *P. incisi* pupae subjected to CS1 and CS3 does not result in significant adverse effects on the emergence rate and quality of post-storage adults. Such information is vital for the mass production and release of *P. incisi* as a dominant biological control agent against *B. dorsalis* in that region of China. In addition, in this study, we used microscopic examination to distinguish parasitized puparia and unparasitized puparia to facilitate scientific research. However, for the mass storage of *P. incisi* pupae for release, we still propose using physical approaches, such as utilizing the suitable mesh of net that prevents flies from escaping, while without restricting the emerged *P. incisi*. Further studies will be carried out to assess the potential control efficacy of post-storage *P. insici* against the *B. dorsalis* population under field conditions.

## Figures and Tables

**Table 1:** Emergence rate of *P. incisi* after being subjected to different pupal cold storage treatments (*n* = 9).

Emergence Rate (%)				
Storage Temperature (℃)	Storage Time (d)	Pupal Age Interval		
		Prepupae	Middle-Age	Late-Age
4	10	7.57 ± 1.66 c	16.84 ± 1.96 b	4.55 ± 1.13 cd
	15	1.77 ± 0.89 ef	6.64 ± 1.50 c	0 f
	20	0 f	1.14 ± 0.76 f	0 f
	25	0 f	0 f	0 f
25 (control)		83.27 ± 2.36 a		
7	10	13.71 ± 2.17 c	25.22 ± 2.74 b	11.47 ± 1.28 c
	15	3.43 ± 1.2 de	11.08 ± 0.87 c	1.53 ± 0.84 de
	20	0.51 ± 0.51 e	5.44 ± 1.63 d	0 e
	25	0 e	0 e	0 e
25 (control)		83.27 ± 2.36 a		
10	10	19.03 ± 2.02 c	45.81 ± 2.50 b	39.07 ± 3.23 b
	15	15.23 ± 2.14 cd	36.19 ± 2.34 b	20.05 ± 2.04 c
	20	9.34 ± 1.56 de	16.29 ± 2.86 cd	3.36 ± 1.11e fg
	25	2.64 ± 0.84 fg	5.46 ± 1.42 ef	0 g
25 (control)		83.27 ± 2.36 a		
13	10	37.89 ± 3.16 c	81.29 ± 2.50 a	80.62 ± 1.92 a
	15	33.52 ± 4.32 cd	73.22 ± 2.34 a	75.71 ± 3.26 a
	20	20.06 ± 3.06 d	54.80 ± 2.73 b	59.33 ± 2.44 b^E^
	25	6.01± 1.48 e	38.49 ± 2.95 c^E^	46.67± 2.64 bc^E^
25 (control)		83.27 ± 2.36 a		

Note: Data are presented as mean ± SE. Different lowercase letters indicate significant differences at the 0.05 level by Tukey’s test. Control means *P. incisi* pupae developed at 25 °C. ^E^ means that part of *P. incisi* have emerged during the cold storage protocol.

**Table 2:** Proportion of emerging *P. incisi* females after being subjected to different pupal cold storage treatments (*n* = 9).

Female Proportion (%)				
Storage Temperature (℃)	Storage Time (d)	Pupal Age Interval		
		Prepupae	Middle-Age	Late-Age
4	10	80.21 ± 7.56 a	69.51 ± 5.06 a	-
	15	-	72.92 ± 11.86 a	-
	20	-	-	-
	25	-	-	-
25 (control)	-	71.53 ± 2.02 a		
7	10	71.48 ± 6.78 a	71.42 ± 4.92 a	74.07 ± 5.97 a
	15	-	64.82 ± 10.09 a	-
	20	-	80.56 ± 7.38 a	-
	25	-	-	-
25 (control)		71.53 ± 2.02 a		
10	10	74.23 ± 6.01 a	68.21 ± 2.96 a	69.81 ± 2.85 a
	15	71.09 ± 6.17 a	71.65 ± 3.06 a	72.67 ± 4.30 a
	20	71.88 ± 8.78 a	70.95 ± 4.15 a	-
	25	-	80.95 ± 8.13 a	-
25 (control)		71.53 ± 2.02 a		
13	10	70.65 ± 5.34 a	69.20 ± 3.08 a	66.79 ± 1.99 a
	15	69.58 ± 4.01 a	70.12 ± 2.62 a	68.43 ± 1.98 a
	20	73.70 ± 5.78 a	69.23 ± 4.06 a	71.56 ± 3.21 a^E^
	25	68.75 ± 15.27 a	70.56 ± 4.26 a^E^	69.42 ± 2.43 a^E^
25 (control)		71.53 ± 2.02 a		

Note: Data are presented as mean ± SE. Different lowercase letters indicate significant differences at the 0.05 level by Tukey’s test. Control means *P. incisi* pupae developed at 25 °C. ^E^ means that part of *P. incisi* have emerged during the cold storage protocol. “-” means that the emergence rate was less than 5% after pupal cold storage, and therefore excluded from the analysis.

**Table 3:** The dry weight of G1 *P. incisi* post-storage females (a) and males (b) that had been subjected to different pupal cold storage treatments (*n* = 9).

Treatment	Dry Weight (mg)		Flight Ability (%)	
	Female	Male	Female	Male
Control (25 °C)	59.67 ± 0.76 a	40.30 ± 0.59 a	69.33 ± 3.65 a	62.67 ± 2.75 a
CS1	59.05 ± 0.88 ab	40.55 ± 0.38 a	67.56 ± 4.15 ab	60.22 ± 2.99 ab
CS2	59.15 ± 0.59 ab	40.10 ± 0.50 ab	60.22 ± 1.61 ab	54.44 ± 2.97 ab
CS3	57.72 ± 0.60 ab	39.24 ± 0.34 ab	61.33 ± 1.91 ab	55.11 ± 2.21 ab
CS4	56.46 ± 0.97 b	38.19 ± 0.64 b	56.67 ± 1.86 b	52.00 ± 1.70 b

Note: CS1: Cold storage 1, late-age pupae stored at 13 °C for 10 d; CS2: cold storage 2, middle-age pupae stored at 13 °C for 10 d; CS3: cold storage 3, late-age late pupae stored at 13 °C for 15 d; CS4: cold storage 4, middle-age pupae stored at 13 °C for 15 d. Control means *P. incisi* pupae developed at 25 °C. Bars topped by the same letter do not differ significantly (*p* > 0.05) according to Tukey’s HSD test (one-way ANOVA).

**Table 4:** Reproductive parameters of G1 *P. incisi* post-storage adults (*n* = 15).

Treatment	Pre-Oviposition Period (d)	Oviposition Period (d)	Post-Oviposition Period (d)	Total No. of Offspring	No. of Daily Offspring
25 °C (control)	0.33 ± 0.13 a	10.73 ± 0.73 a	2.73 ± 0.85 a	69.13 ± 4.44 a	6.72 ± 0.41 a
CS1	0.27 ± 0.15 a	11.07 ± 0.72 a	2.53 ± 0.56 a	68.27 ± 3.75 ab	6.28 ± 0.45 ab
CS2	0.20 ± 0.11 a	9.60 ± 0.66 ab	1.60 ± 0.42 a	54.80 ± 5.51 b	5.53 ± 0.37 ab
CS3	0.27 ± 0.12 a	10.40 ± 0.58 ab	2.00 ± 0.48 a	65.60 ± 3.84 ab	6.46 ± 0.31 a
CS4	0.40 ± 0.13 a	7.73 ± 0.67 b	0.73 ± 0.25 a	37.07 ± 4.59 c	4.92 ± 0.40 b

Note: Data are presented as mean ± SE. Different lowercase letters indicate significant differences at the 0.05 level by Tukey’s test. CS1: cold storage 1, late-age pupae stored at 13 °C for 10 d; CS2: cold storage 2, middle-age pupae stored at 13 °C for 10 d; CS3: cold storage 3, late-age late pupae stored at 13 °C for 15 d; CS4: cold storage 4, middle-age pupae stored at 13 °C for 15 d. Control means *P. incisi* pupae developed at 25 °C.

**Table 5:** Emergence rate and female proportion of progeny (G2) (*n* = 15).

Treatment	G2 Emergence Rate (%)	G2 Female Proportion (%)
Control (25 °C)	85.51 ± 1.31 a	71.36 ±1.44 a
CS1	83.75 ± 1.20 a	69.43 ±1.13 a
CS2	83.45 ± 1.52 a	70.73 ± 0.95 a
CS3	83.96 ± 1.11 a	71.02 ± 0.73 a
CS4	84.23 ± 1.21 a	67.26 ± 1.58 a

Note: Data are presented as mean ± SE. Different lowercase letters indicate significant differences at the 0.05 level by Tukey’s test. CS1: cold storage 1, late-age pupae stored at 13 °C for 10 d; CS2: cold storage 2, middle-age pupae stored at 13 °C for 10 d; CS3: cold storage 3, late-age late pupae stored at 13 °C for 15 d; CS4: cold storage 4, middle-age pupae stored at 13 °C for 15 d. Control means *P. incisi* pupae developed at 25 °C.

## Data Availability

The data presented in this study are available from the corresponding author on reasonable request.

## References

[1] Liu H., Zhang D.J., Xu Y.J., Wang L., Cheng D.F., Qi Y.X., Zeng L., Lu Y.Y. (2019). Invasion, expansion, and control of *Bactrocera dorsalis* (Hendel) in China. J. Integr. Agric..

[2] Clarke A.R., Li Z.H., Qin Y.J., Zhao Z.H., Liu L.J., Schutze M.K. (2019). *Bactrocera dorsalis* (Hendel) (Diptera: *Tephritidae*) is not invasive through Asia: It’s been there all along. J. Appl. Entomol..

[3] Mutamiswa R., Nyamukonduwa C., Chikowore G., Chidawanyika F. (2021). Overview of oriental fruit fly, *Bactrocera dorsalis* (Hendel) (Diptera: *Tephritidae*) in Africa: From invasion, bio-ecology to sustainable management. Crop. Prot..

[4] Piñero J.C., Souder S.K., Smith T.R., Vargas R.I. (2017). Attraction of *Bactrocera cucurbitae* and *Bactrocera dorsalis* (Diptera: *Tephritidae*) to beer waste and other protein sources laced with ammonium acetate. Fla. Entomol..

[5] Jin T., Zeng L., Lin Y.Y., Lu Y.Y., Liang G.W. (2011). Insecticide resistance of the oriental fruit fly, *Bactrocera dorsalis* (Hendel) (Diptera: *Tephritidae*), in mainland China. Pest. Manag. Sci..

[6] Diaz-Fleischer F., Perez-Staples D., Cabrera-Mireles H., Montoya P., Liedo P. (2017). Novel insecticides and bait stations for the control of *Anastrepha* fruit flies in mango orchards. J. Pestic. Sci..

[7] Yang J.Q., Cai P.M., Chen J., Zhang H.H., Wang C., Xiang H.J., Yang Y.C., Chen J.H., Ji Q.E., Song D.B. (2018). Interspecific competition between Fopius arisanus and Psyttalia incisi (Hymenoptera: *Braconidae*), parasitoids of Bactrocera dorsalis (Diptera: *Tephritidae*). Biol. Control.

[8] Liang G.H., Chen J.H., Huang J.C. (2006). The functional response and disturbance response of larvae of *Psyttalia incisi* (Silvestri) to oriental fruit flies. Acta Agric. Univ. Jiangxiensis.

[9] Liang G.H., Wu Y., Chen J.H. (2006). Seasonal incidence of *Bactrocera dorsalis* and its parasitoids in field. J. Southwest For. Coll..

[10] Colinet H., Boivin G. (2011). Insect parasitoids cold storage: A comprehensive review of factors of variability and consequences. Biol. Control.

[11] Rathee M., Ram P. (2018). Impact of cold storage on the performance of entomophagous insects: An overview. Phytoparasitica.

[12] Rezaei M., Talebi A.A., Fathipour Y., Karimzadeh J., Mehrabadi M., Reddy G.V.P. (2020). Effects of cold storage on life-history traits of *Aphidius matricariae*. Entomol. Exp. Appl..

[13] Ghazy N.A., Suzuki T., Amano H., Ohyama K. (2014). Air temperature optimisation for humidity-controlled cold storage of the predatory mites *Neoseiulus californicus* and *Phytoseiulus persimilis* (Acari: *Phytoseiidae*). Pest. Manag. Sci..

[14] Lü X., Han S.C., Li J., Liu J.S., Li Z.G. (2019). Effects of cold storage on the quality of *Trichogramma dendrolimi* Matsumura (*Hymenoptera*: *Trichogrammatidae*) reared on artificial medium. Pest. Manag. Sci..

[15] Daane K.M., Wang X.G., Johnson M.W., Cooper M.L. (2013). Low temperature storage effects on two olive fruit fly parasitoids. BioControl.

[16] Long X.Z., Chen K.W., Xian J.D., Lu Y.Y., Zeng L. (2014). Cold storage technique of *Diachasmimorpha longicaudata* (Ashmead). J. Environ. Entomol..

[17] Wang H.L. (2011). Effects of Spinosad and Temperature and Humidity on the Parasitoid *Fopius arisanus* (Sonan). Master’s Thesis.

[18] Ji Q.E., Dong C.Z., Chen J.H. (2004). A new record species—*Opius incisi* Silvestri (*Hymenoptera*: *Braconidae*) parasitizing on *Dacus dorsalis* (Hendal) in China. Entomotaxonomia.

[19] Chang C.L., Vargas R.I., Caceres C., Jang E., Cho I.K. (2006). Development and assessment of a liquid larval diet for *Bactrocera dorsalis* (Diptera: *Tephritidae*). Ann. Entomol. Soc. Am..

[20] Wang X.G., Messing R.H. (2004). Potential interactions between pupal and egg-or larval-pupal parasitoids of Tephritid fruit flies. Environ. Entomol..

[21] Vargas R.I., Leblanc L., Harris E., Manoukis N.C. (2012). Regional suppression of *Bactrocera* fruit flies (Diptera: *Tephritidae*) in the Pacific through biological control and prospects for future introductions into other areas of the world. Insects.

[22] Yao M.J., Xie C.H., He Y.B., Qiu B., Chen H.Y., Xu Z.F. (2008). Investigation on hylmenopterous parasitoids of *Bactrocera dorsalis* (Hendel) in Guangdong. J. Environ. Entomol..

[23] Benelli M., Ponton F., Lallu U., Mitchell K.A., Taylor P.W. (2019). Cool storage of Queensland fruit fly pupae for improved management of mass production schedules. Pest. Manag. Sci..

[24] Sakaki S., Jalali M.A., Kamali H., Nedved O. (2019). Effect of low-temperature storage on the life history parameters and voracity of *Hippodamia variegata* (Coleoptera: *Coccinellidae*). Eur. J. Entomol..

[25] Benelli M., Ponton F., Taylor P.W. (2019). Cool sotrage of Queensland fruit fly eggs for increased flexibility in rearing programs. Pest. Manag. Sci..

[26] Luczynski A., Nyrop J.P., Shi A. (2006). Influence of cold storage on pupal development and mortality during storage and on post-storage performance of *Encarsia formosa* and *Eretmocerus eremicus* (Hymenoptera: Aphelinidae). Biol. Control.

[27] Neven L.G., Hansen L.D. (2010). Effects of temperature and controlled atmospheres on codling moth metabolism. Ann. Entomol. Soc. Am..

[28] Lins J.C., Bueno V.H.P., Sidney L.A., Silva D.B., Sampaio M.V., Pereira J.M., Nomelini Q.S.S., van Lenteren J.C. (2013). Cold storage affects mortality, body mass, lifespan, reproduction and flight capacity of *Praon volucre* (*Hymenoptera*: *Braconidae*). Eur. J. Entomol..

[29] Jervis M.A., Ferns P.N., Heimpel G.E. (2003). Body size and the timing of egg production in parasitoid wasps: A comparative analysis. Funct. Ecol..

[30] Colinet H., Boivin G., Hance T. (2007). Manipulation of parasitoid size using the temperature-size rule: Fitness consequences. Oecologia.

[31] Visser B., le Lann C., den Blanken F.J., Harvey J.A., van Alphen J.M., Ellers J. (2010). Loss of lipid synthesis as an evolutionary consequence of a parasitic lifestyle. Proc. Natl. Acad. Sci. USA.

[32] Ismail M., van Baaren J., Hance T., Perre J.S., Vernon P. (2013). Stress intensity and fitness in the parasitoid *Aphidius ervi* (*Hymenoptera*: *Braconidae*): Temperature below the development threshold combined with a fluctuating thermal regime is a must. Ecol. Entomol..

[33] Alam M.S., Alam M.Z., Alam S.N., Miah M.R.U., Mian M.I.H. (2016). Effect of storage duration on the stored pupae of parasitoid *Bracon hebetor* (Say) and its impact on parasitoid quality. Bangladesh J. Agric. Res..

[34] Amice G., Vernon P., Outreman Y., van Alphen J., van Baaren J. (2008). Variability in responses to thermal stress in parasitoids. Ecol. Entomol..

[35] Yocum G.D., Zdárek J., Joplin K.H., Lee R.E., Smith D.C., Manter K.D., Denlinger D.L. (1994). Alteration of the eclosion rhythm and eclosion behavior in the flesh fly, Sarcophaga crassipalpis, by low and high temperature stress. J. Insect. Physiol..

[36] Yan Z., Yue J.J., Bai C., Peng Z.Q., Zhang C.H. (2017). Effects of cold storage on the biological characteristics of *Microplitis prodeniae* (*Hymenoptera*: *Braconidae*). Bull. Entomol. Res..

[37] Vargas R.I., Ramadan M., Hussain T., Mochizuki N., Bautista R.C., Stark J.D. (2002). Comparative demography of six fruit fly (Diptera: *Tephritidae*) parasitoids (*Hymenoptera*: *Braconidae*). Biol. Control.

[38] Liang G.H., Huang J.C., Chen J.H. (2007). Comparative analysis on RAPD of two geographic populations of *Psyttalia incisi*. J. Fujian Coll. For..

[39] Leopold R., Vreysen M.J.B., Robinson A.S., Hendrichs J. (2007). Colony maintenance and mass-rearing: Using cold storage technology for extending the shelf-life of insects. Area-Wide Control of Insect Pests: From Research to Field Implementation.

